# Role of serum biomarkers in predicting management strategies for acute pulmonary embolism

**DOI:** 10.1016/j.heliyon.2023.e21068

**Published:** 2023-10-17

**Authors:** Hadeer Ahmed Elshahaat, Niveen E. Zayed, Marwa Abdel-monem Ateya, Mohamed Safwat, Amr Talaat El Hawary, MohammedM.N. Abozaid

**Affiliations:** a Chest Department, faculty of Medicine of Zagazig University, Zagazig, Egypt; b Clinical Pathology Department, Faculty of medicine of Zagazig University, Zagazig, Egypt; c Cardiology Department, Faculty of medicine of Zagazig University, Zagazig, Egypt; d Internal Medicine Department, Faculty of medicine of Zagazig University, Zagazig, Egypt

**Keywords:** Pulmonary embolism, Risk stratification, Biomarkers

## Abstract

**Background:**

Acute pulmonary embolism (APE) is a condition that can be fatal. The severity of the disease influences therapeutic decisions, and mortality varies significantly depending on the condition's severity. Identification of patients with a high mortality risk is crucial. Since inflammation, hemostatic, and coagulation abnormalities are linked to APE, serum biomarkers may be helpful for prognostication.

**Aim:**

To evaluate the significance of serum biomarkers in APE risk assessment and the suitability of these biomarkers for management and decision-making.

**Methods:**

This study involved 60 adult patients with APE who were divided according to risk categorization. It was conducted in Chest, Cardiology and Internal Medicine department, Zagazig University Hospitals from December 2022 to May 2023. Several hematological biomarkers and their significance in APE risk assessment were measured with a comparison with the latest risk stratification methods which include haemodynamic measures and right ventricular (RV) dysfunction echocardiographic markers.

**Results:**

Each risk group involved 20 patients (high, intermediate (10 were intermediate-high and 10 were intermediate-low) and low risk group). They were 34 females and 26 males with the mean ± SD of their age was 59.25 ± 13.06 years. Regarding hematological biomarkers, there were statistically significant differences as regards; lymphocytes, platelet to lymphocyte ratio (PLR), albumin, blood urea nitrogen (BUN), C-reactive protein (CRP) and D-dimer with highly statistically significant differences as regards; neutrophil to lymphocyte ratio (NLR), BUN to albumin (B/A) ratio, troponin I (TnI), and brain natriuretic peptide (BNP). TnI had the highest specificity and predictive value positive (PVP) and BNP had the highest sensitivity and predictive value negative (PVN) in predicting high risk groups. The Lymphocyte and NLR showed the lowest sensitivity and the albumin and B/A ratio had the lowest specificity. Regarding transthoracic echocardiography (TEE); there was a statistically significant increase regarding pulmonary artery systolic pressure (PASP) and a highly statistically significant increase regarding the right ventricle/left ventricle (RV/LV) ratio. There were statistically significant decreases regarding tricuspid annular plane systolic excursion (TAPSE) and peak systolic velocity of tricuspid annulus (S′) among risk groups.

**Conclusion:**

APE prognosis can be judged accurately by simultaneously measuring a few biomarkers along with haemodynamic variables and echocardiographic parameters of RV dysfunction.

## 1 Introduction

Acute pulmonary embolism (APE) is a serious cardiovascular condition that is associated with greater mortality and morbidity incidence. APE follows acute myocardial infarction and stroke as the third most prevalent cardiovascular reason for death [1]. Clinical manifestation, physical assessment, imaging investigations, and serum biomarkers are some of the variables linked to a poor prognosis [2].

A high prevalence of clinical misdiagnosis and missed diagnosis occurs in APE patients, the majority of whom have no symptoms or vague signs. Patients can start receiving effective therapy directly after they have been appropriately detected in the early stages. As a result, rapid exact diagnosis and prompt risk assessment of APE patients are crucial for effective treatment and improved consequences [3].

The Pulmonary Embolism Severity Index (PESI) and its simplified variant (sPESI), which is frequently used to stratify patients by mortality risk and are proficient at detecting low-risk individuals appropriate for outpatient care, are the most commonly utilised prognostic risk indicators. However, due to their limited specificity, these models' accuracy is unsatisfactory for patients at higher risk, and additional investigations may be time- and resource-intensive and expensive for the patients [4].

At the time of hospital admission, in shocked APE group (high risk), the death rate is close to 50 %, and the latest recommendations advise thrombolytics for them. Of all APE patients, approximately 90 % are normotensive, however, they have a very diverse short-term mortality rate ranging from 1 % to 15 %. The best way to treat this group, anticoagulation or more forceful measures, is still up for dispute [5].

There is still a necessity for a prognostic tool that can classify a subgroup of patients who are stable hemodynamically but have a high chance of having unfavorable consequences. “Poor outcomes” makes this more challenging. Physicians could better direct treatment based on each patient's risk if they could anticipate normotensive patients with bad prognosis [6].

The right ventricular (RV) dysfunction noticed by the echocardiogram is a helpful indicator of risk assessment in APE; nonetheless, it has modest positive predictive value for expecting short term mortality and it is unable to distinguish between patients who require close monitoring and those who can be managed as outpatients [7].

To diagnose and categorize APE patients according to risk, numerous biomarkers have been proposed. The majority of them were selected from the immune reaction process, tissues, and organs, as well as the coagulation-fibrinolysis pathways [3].

## 2 Aim of the study

To evaluate the significance of serum biomarkers in APE risk assessment and the suitability of these biomarkers for management and decision-making.

## 3 Materials and methods

### 3.1 Technical design

#### 3.1.1 The population of the study

This study involved 60 adult patients with APE (20 patients in each group high, intermediate (10 patients were intermediate-high and 10 patients were intermediate-low) and low risk groups). They were 34 females and 26 males with the mean ± SD of their age was 59.25 ± 13.06 years.

3.1.2. Inclusion criteria: Adult patients (≥18 years old) who were confirmed diagnosis of acute pulmonary embolism according to the European Society of Cardiology (ESC) guidelines **(ESC, 2019):** [8].

- A computed tomography pulmonary angiography (CTpA) is used to confirm a PE when a thrombus is directly visible in two projections, in the form of filling defect or amputated branch of pulmonary artery.

#### 3.1.2 Exclusion criteria

Patients were excluded if they.

(1) Received thrombolytic or anticoagulant prior to their blood investigation.
(2) Encountered one of the subsequent disorders: a) blood disorders; b) patients with proved recent infection; c) received blood transfusion within the former two weeks; d) received corticosteroid or immunosuppressive medications through the previous two weeks; e) patients with acute coronary syndrome, myocardial disorder, left ventricular dysfunction, myocarditis, chronic pulmonary hypertension, chronic obstructive pulmonary disease; e) progressive hepatic or renal disorders; f) acute or chronic inflammatory disorders as vasculitis, rheumatoid arthritis, systemic lupus erythematosus; g) acute ischemic cerebrovascular disorder, acute mesenteric ischemia or acute peripheral arterial occlusion.

3.1.4. Study design: This study was comparative cross sectional study that conducted in Chest, Cardiology, and Internal Medicine Department, Zagazig University Hospitals for 6 months from December 2022 to May 2023.

### 3.2 Operational design

To the patients, the study was thoroughly explained. Informed and written consent was provided by each patient. The following was done for our patients.

- The patients' demographics and complete medical histories were recorded.
- Full clinical examination & vital signs were evaluated.
- Clinical prediction rules were assessed by Wells role and patients were categorized to; low, intermediate, high clinical probability (0–1, 2–6,>6 respectively) of APE [9]**.**
- Electrocardiography (ECG): ECG variations suggestive of RV strain in more severe cases of APE as; T-wave inversion in V1–V4 leads, QR pattern in V1, S1Q3T3 pattern, and right bundle branch block. Sinus tachycardia is the only defect in milder conditions [10]**.**
- Hematological biomarkers: venous blood samples were aseptically withdrawn by venepuncture within 3 h of admission to estimate:
1 Routine investigations:
a) Two ml of the blood sample was delivered into a sterile vacutainer containing EDTA for complete blood cell counts (CBC) examination using XN 2000 automatic cell counter (Sysmex, Japan). The ratio of neutrophil count to lymphocyte count (NLR) and platelet-lymphocyte ratio (PLR), and hematocrit to hemoglobin ratio (HHR) were calculated
b) 2.7 ml was delivered into a sterile vacutainer tube containing trisodium citrate for:
- Coagulation testing (PT, PTT, and INR): operated on automated blood coagulation analyzer, model CS 2500 (Sysmex, Japan).
- D-dimer estimation was carried out by immunoturbidimetric methodology utilising manufacturer-supplied reagent on a Roche Cobas 6000 autoanalyzer (c501 molecule) (Roche diagnostics, Switzerland), its upper normal limit is 500 ng/mL.
c) 3 ml were delivered into a sterile plain vacutainer tube centrifuged for 15 min for serum separation used to measure:
- Liver and kidney function tests (LFT, KFT) operated spectrophotometry using a manufacturer-supplied kit on Roche Cobas 8000, c702 module (Roche diagnostics, Switzerland). The B/A ratio (mg/g) (blood urea nitrogen (BUN) (mg/dL) divide serum albumin (g/dL)) were calculated.
- C- reactive protein (CRP) operated on Roche Cobas 6000 autoanalyzer (c501 molecule) (Roche diagnostics, Switzerland). The upper limit of normal 5 mg/L.
- Troponin I (TnI) operated by e−411. The normal range of is 0–0.1 ng/mL.
2 Special laboratory techniques: 2 ml were withdrawn into a sterile plain vacutainer tube centrifuged for 15 min for serum separation and stored at −20 till measurement of the following markers by Enzyme linked Immunosorbent assay (ELISA) and all were performed according to manufacture instruction:
- Brain natriuretic peptide (BNP): catalogue No EHFABP3 (thermofisher). The normal range of BNP was <100 pg/mL
- Heart-type fatty acid -binding protein (H-FABP): catalogue No EHNPPB. H-FABP concentration ≥6 ng/mL was related with a bad short-term consequence.
- Ischemia modified albumin (IMA): catalogue No abx258294 (abbexa).
- Transthoracic Echocardiography (TTE) was performed by **(Sonoscape SSI-4000)** and documented within 24 h after admission by a qualified echocardiographer. Signs of RV overload and/or dysfunction are RV dilatation with basal RV/LV proportion more than 1.0, impaired free wall contractility in comparison to its apex (McConnell sign), reduced tricuspid annular plane systolic excursion (TAPSE) measured with M-Mode (<16 mm), and reduced peak systolic (S′) velocity of tricuspid annulus (<9.5 cm/s). Pulmonary artery systolic pressure (PASP) was measured [8]**.**
- Computed tomography pulmonary angiography (CTpA), to approve APE diagnosis and identify RV enlargement as a sign of RV dysfunction (RV end-diastolic diameter and RV/LV proportion in the transverse or 4-chamber imaging) [11]**.**
- The categorization of APE severity and early mortality was estimated according to **ESC, 2019** based on 4 factors; hemodynamic instability, pulmonary embolism severity index (PESI) class III-V or simplified PESI (sPESI) ≥ 1, RV dysfunction by TTE or CTpA and raised troponin; and patients were categorized to; high **(d)**, intermediate high **(c)**, intermediate low **(b)** and low **(a)** risk [8]**.**
- All patients started standard anticoagulant using subcutaneous body weight-adjusted doses of low molecular weight heparin or intravenous unfractionated heparin. Thrombolysis is indicated in high-risk APE. Initially stable patients who experienced hemodynamic worsening were also recommended thrombolysis [8]**.**
- Outcomes were recorded:
- Improved: patients became clinically and vitally stable.
- Deteriorated: patients arrested, became hemodynamically unstable, or needed mechanical ventilation.
- Death.

### 3.3 Administrative design

agreement was obtained from Zagazig university institution review board **(ZU-IRB #10144/28**–**12**–**2022)**

### 3.4 Statistical analysis

Using SPSS version 19, all information was gathered, tabulated, and statistically assessed. Categorical qualitative parameters were presented as absolute frequencies (number) and relative frequencies (%), while continuous quantitative parameters were presented as the mean ± SD. Utilising the Shapiro-Wilk test, continuous data were analyzed for normality. More than two sets of properly distributed data were compared using one way ANOVA (F test). Each of the two groups was compared separately using the Least Significant Difference post hoc test (LSD). Fisher exact test and the Chi-square test were used to compare data that was categorical.

Each test had two sides. P-values <0.05 were classified as statistically significant (S), p-values <0.001 as highly statistically significant (HS), and p-values ≥0.05 as statistically insignificant (NS). To assess the test's capacity to discriminate between risk groups, a receiver-operator characteristic (ROC) curve was used.

## 4 Results

60 patients were enrolled in our study and were divided into 20 patients in each group: high risk, intermediate risk [10 patients were intermediate-high and 10 patients were intermediate-low] and low risk group according to ESC, 2019 [8]. Low risk group (a) were 12 females (60 %) and 8 males (40 %) with a mean ± SD age of 53.5 ± 15 years. Intermediated-low group (b) were 5 females (50 %) and 5 males (50 %) with a mean ± SD age of 58.2 ± 9.4 years. Intermediate-high group (c) were 6 females (60 %) and 4 males (40 %) with a mean ± SD age of 61.4 ± 12.7 years. High risk group were 11 females (55 %) and 9 males (45 %) with a mean ± SD age of 64.4 ± 11.2 years.

Table 1 showed the comparison of demographic characteristics and vital signs of all studied groups as regards age, sex, body mass index (BMI), smoking and comorbidities. There were statistically non-significant differences between the risk groups. There were statistically significant differences as regards heart rate and SPO_2_ (P = 0.005, 0.002 respectively) and highly statistically significant differences as regards systolic blood pressure (SBP) and diastolic blood pressure (DBP) (P < 0.001).

**Table 1:** Demographic characteristics and vital signs of all study groups (n = 60).

Variable	Low risk Group (a) (n = 20)	Intermediate low risk Group (b) (n = 10)	Intermediate high risk Group (c) (n = 10)	High risk Group (d) (n = 20)	P
**Age: (years)** Mean ± SD	53.5 ± 15	58.2 ± 9.4	61.4 ± 12.7	64.4 ± 11.2	0.06 (NS)
**BMI (kg/m**^**2**^**):** Mean ± SD	30.7 ± 5.9	31.4 ± 6.3	30.7 ± 8.1	31.1 ± 6.8	0.99 (NS)
**Sex:** Male (26): Female (34):	8 (40 %) 12 (60 %)	5 (50 %) 5 (50 %)	4 (40 %) 6 (60 %)	9 (45 %) 11 (55 %)	0.95 (NS)
**Smoking:** No: Yes:	16 (80 %) 4 (20 %)	8 (80 %) 2 (20 %)	7 (70 %) 3 (30 %)	14 (70 %) 6 (30 %)	0.85 (NS)
**Comorbidities:** Asthma: DM: HPN: DM/HPN: No:	1 (5 %) 4 (20 %) 3 (15 %) 1 (5 %) 11 (55 %)	0 (0 %) 2 (20 %) 2 (20 %) 0 (0 %) 6 (60 %)	0 (0 %) 1 (10 %) 2 (20 %) 1 (10 %) 6 (60 %)	1 (5 %) 3 (15 %) 4 (20 %) 1 (5 %) 11 (55 %)	0.98 (NS)
**Heart rate (bpm):** Mean ± SD	a,b,c 89.5 ± 12.6	a,b,c 94.6 ± 11.8	a,b,c,d 98.5 ± 16.1	c,d 105.2 ± 13.5	**0.005 (S)**
**SBP (mmHg):** Mean ± SD	a,b 127.5 ± 16.1	a,b 128.5 ± 14.9	c 114.5 ± 17.8	d 85 ± 13.2	**<0.001 (HS)**
**DBP (mmHg):** Mean ± SD	a,b,c 80.2 ± 11.7	a,b,c 80.5 ± 10.3	a,b,c 73 ± 10.5	d 53.2 ± 9.1	**<0.001 (HS)**
**SpO**_**2**_**:** Mean ± SD	a,b,c 92.6 ± 3.4	a,b,c 91.5 ± 4.5	a,b,c,d 90.3 ± 3.4	c,d 87.2 ± 5	**0.002 (S)**

Data expressed as frequency (percentage), mean and (SD).

*NS* non-significant difference (p > 0.05), *S* statistically significant (P < 005), *HS* Highly statistically significant (P < 0.001), Different letters denote significant difference.

*SBP* systolic blood pressure, *DBP* Diastolic blood pressure, *SpO*_*2*_: oxygen saturation.

Comparison of hematological biomarkers according to APE risk groups is presented in Table 2. As regards CBC, there were statistically non-significant differences among risk groups as regards total leucocytic count (TLC), neutrophils, hemoglobin, hematocrit, platelet (PLT), and HHR (P value = 0.99, 0.59, 0.56, 0.98, 0.98, and, 0.21 respectively) while statistically significant regarding lymphocytes and PLR (P = 0.001, 0.005 respectively) and highly statistically significant increase regarding NLR (P < 0.001).

**Table 2:** Hematological biomarkers according to risk groups (n = 60).

Variable	Low risk Group (a) (n = 20)	Intermediate low risk Group (b) (n = 10)	Intermediate high risk Group (c) (n = 10)	High risk group (d) (n = 20)	P
**TLC(10³/ul):** Mean ± SD	8.3 ± 2.1	8.3 ± 2.7	8.4 ± 2.4	8.5 ± 2.5	0.99 (NS)
**Neutrophils (10³/ul):** Mean ± SD	7.6 ± 1.9	8.1 ± 2.2	8.2 ± 2.9	8.6 ± 2.3	0.59 (NS)
**Lymphocytes (10³/ul):** Mean ± SD	a,b 1.7 ± 0.6	a,b,c,d 1.3 ± 0.6	b,c,d 1.1 ± 0.5	b,c,d 0.9 ± 0.4	**0.001 (S)**
**NLR:** Mean ± SD	a,b 4.9 ± 2.4	a,b 6.8 ± 2.6	c,d 8.4 ± 4.2	c,d 10 ± 3.9	**<0.001 (HS)**
**Hemoglobin (g/dl):** Mean ± SD	13.1 ± 1.7	13.8 ± 1.5	12.7 ± 2.1	13.2 ± 1.8	0.56 (NS)
**Hematocrit (%):** Mean ± SD	37.4 ± 7.4	37.6 ± 6.6	36.8 ± 8.4	36.7 ± 6.9	0.985 (NS)
**HHR:** Mean ± SD	2.8 ± 0.2	2.6 ± 0.2	2.8 ± 0.2	2.7 ± 0.2	0.213 (NS)
**PLT(10³/ul):** Mean ± SD	257.1 ± 73	253.1 ± 95	249.7 ± 91	247.2 ± 70	0.98 (NS)
**PLR:** Mean ± SD	a,b 171.1 ± 65	a,b,c 205.8 ± 76	b,c,d 228.6 ± 84	b,c,d 262.7 ± 85	**0.005 (S)**
**Albumin (g/dl):** Mean ± SD	a,b 3.9 ± 0.7	a,b,c 3.7 ± 0.8	b,c,d 3.2 ± 0.6	c,d 3.1 ± 0.5	**0.001 (S)**
**ALT(U/L):** Mean ± SD	25.4 ± 10	32.3 ± 20	29.8 ± 21	23.2 ± 9	0.39 (NS)
**AST(U/L):** Mean ± SD	28.9 ± 11.7	35.4 ± 29.2	31.3 ± 11.5	31.9 ± 21.8	0.86 (NS)
**BUN(mg/dl):** Mean ± SD	a,b,c 18.2 ± 5.7	a,b,c 20.7 ± 13.5	a,b,c,d 25.1 ± 9	c,d 29.9 ± 10.3	**0.002 (S)**
**Creatinine (mg/dl):** Mean ± SD	0.79 ± 0.2	0.81 ± 0.5	0.84 ± 0.3	0.81 ± 0.3	0.99 (NS)
**B/A (mg/g):** Mean ± SD	a,b 4.7 ± 1.5	a,b,c 5.2 ± 2.4	b,c,d 7.8 ± 2.6	c,d 10.1 ± 4	**<0.001 (HS)**
**PT(Sec):** Mean ± SD	13.3 ± 1.9	14.5 ± 2.2	13.8 ± 1.8	14.8 ± 2.1	0.12 (NS)
**PTT(Sec):** Mean ± SD	31.5 ± 4.1	30.1 ± 5.8	30.7 ± 5.1	30.4 ± 0.9	0.83 (NS)
**INR:** Mean ± SD	1.06 ± 0.1	1.1 ± 0.1	1.1 ± 0.1	1.1 ± 0.1	0.38 (NS)
**CRP(mg/l):** Mean ± SD	a,b 6.6 ± 4	a,b,c 8.9 ± 4.8	b,c 11.4 ± 4.6	d 13.5 ± 7.1	**0.002 (S)**
**Troponin I(ng/ml):** Mean ± SD	a,b 0.09 ± 0.06	a,b 0.1 ± 0.05	c 0.7 ± 0.4	d 1.4 ± 0.6	**<0.001 (HS)**
**BNP (pg/ml):** Mean ± SD	a,b 125.3 ± 40.5	a,b,c 162.7 ± 67.8	b,c 237 ± 57.3	d 416.5 ± 141.3	**<0.001 (HS)**
**HFABP(ng/ml)** Negative (14): Positive (≥6) (46):	7 (35 %) 13 (65 %)	3 (30 %) 7 (70 %)	2 (20 %) 8 (80 %)	2 (10 %) 18 (90 %)	0.28 (NS)
**D-Dimer (ng/ml):** Mean ± SD	a,b 683 ± 132.5	a,b,c 786 ± 198.2	b,c,d 909 ± 270	c,d 1003 ± 353.2	**0.002 (S)**
**IMA (ng/ml):** Mean ± SD	2189 ± 724	2215 ± 905	2309 ± 770	2388 ± 776	0.86 (NS)

Data represented as frequency (percentage), mean, and (SD).

*NS* non-significant difference (p > 0.05), *S* statistically significant (P < 005), *HS* Highly statistically significant (P < 0.001), Different letters denote significant difference.

*TLC* Total leucocyte count, *NLR* neutrophil/lymphocyte ratio, *HHR* hematocrit to hemoglobin ratio*, PLT* platelet, *PLR* platelet/lymphocyte ratio, *ALT* Alanine transaminase, *AST* Aspartate aminotransferase, *BUN* blood urea nitrogen, *B/A* BUN/albumin ratio, *PT* prothrombin time, *PTT* partial thromboplastin time, *INR* international normalized ratio, *CRP* C-reactive protein, *BNP* Brain natriuretic peptide, *HFABP* Heart type fatty acid binding protein, *IMA* ischemia modified albumin.

Likewise, we compared LFT and KFT between risk groups and resulted that there were statistically non-significant differences among risk groups as regards alanine transaminase (ALT), aspartate aminotransferase (AST) and s.creatinine (P value = 0.39, 0.86 and 0.99 respectively) but regarding s.albumin and BUN, the differences were statistically significant (p value = 0.001and 0.002). B/A ratio values were shown to be highly statistically significant increase between risk groups (P value < 0.001).

According to cardiac biomarkers; our results demonstrated highly statistically significant increase regarding TnI and BNP among APE risk groups (P value < 0.001), otherwise no significant difference as regards HFABP positive cases (p value = 0.28). Additionally, CRP, D-dimer and IMA values were measured and compared between risk groups and our results revealed significant increase in CRP and D-dimer among risk groups (p value = 0.002). Regarding IMA, it showed elevation in its values between risk groups but with no significant difference (P value = 0.86).

Table 3 demonstrated TTE findings among APE risk groups as regards signs of RV dysfunction. Our results proved statistically significant increase among PE risk groups regarding PASP and RV/LV ratio (P value = 0.003, <0.001 respectively). Also, it showed highly statistically significant decrease as regards TAPSE and S' (P value < 0.001).

**Table 3:** TTE findings according to risk groups (n = 60).

Variable	Low risk group (n = 20)	Intermediate low risk group (n = 10)	Intermediate high risk group (n = 10)	High risk group (n = 20)	P
**RV/LV ratio:** Mean ± SD	a,b 0.9 ± 0.08	a,b 0.9 ± 0.05	c,d 1.1 ± 0.05	c,d 1.1 ± 0.05	**<0.001 (HS)**
**PASP (mmHg):** Mean ± SD	a 33.2 ± 3.9	a,b 36.1 ± 4.5	b,c,d 37.3 ± 3.5	b,c,d 38.5 ± 4.9	**0.003 (S)**
**TAPSE (mm):** Mean ± SD	a 23.3 ± 2.7	b 21.4 ± 2.1	c,d 17.1 ± 2.1	c,d 15.3 ± 1.6	**<0.001 (HS)**
**S’(cm/sec):** Mean ± SD	a,b 14.2 ± 3.7	a,b 12.3 ± 2.2	c,d 9.1 ± 1.7	c,d 8.8 ± 1.2	**<0.001 (HS)**

Data represented as mean, and (SD).

*S* statistically significant (P < 005), *HS* Highly statistically significant (P < 0.001), Different letters denote significant difference.

*TTE* transthoracic echocardiography*, RV/LV* right ventricle/left ventricle ratio, *PASP* pulmonary artery systolic pressure, *TAPSE* Tricuspid Annular Plane Systolic Excursion, *S′* peak systolic velocity of tricuspid annulus.

In Table 4, we compared outcomes of our studied patients according to their risk groups. 3 (15 %) patients died, 1 (5 %) patient deteriorated and 16 (80 %) patients improved in high risk group patients. 1 patient (10 %) died, 3 (30 %) patients deteriorated and 6 (60 %) patients improved in intermediate-high risk group patients. In intermediate-low risk group, 2 (20 %) patients deteriorated and 8 (80 %) patients improved with no deaths. 2 (10 %) patients deteriorated and 18 (90 %) patients improved with no deaths among low risk group patients. No statistically significant differences among risk groups as regards outcomes.

**Table 4:** Comparison outcome according to risk groups (n = 60).

Outcome:	Low risk group (n = 20)	Intermediate low risk group (n = 10)	Intermediate high risk group (n = 10)	High risk group (n = 20)	P
Death:	0 (0 %)	0 (0 %)	1 (10 %)	3 (15 %)	0.21 (NS)
Deterioration:	2 (10 %)	2 (20 %)	3 (30 %)	1 (5 %)	0.08 (NS)
Improved:	18 (90 %)	8 (80 %)	6 (60 %)	16 (80 %)	0.24 (NS)

Data represented as frequency (percentage).

*NS* non-significant difference (p > 0.05).

**Fig. 1:**
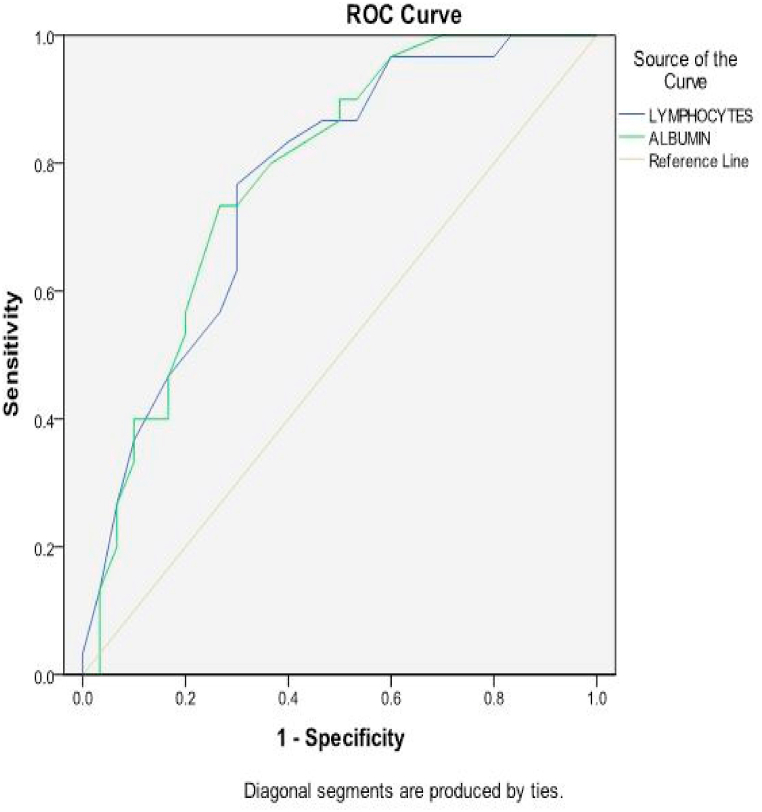
Receiver-operating characteristics (ROC) curve of lymphocytes and albumin in predicting high risk groups among studied patients.

**Fig. 2:**
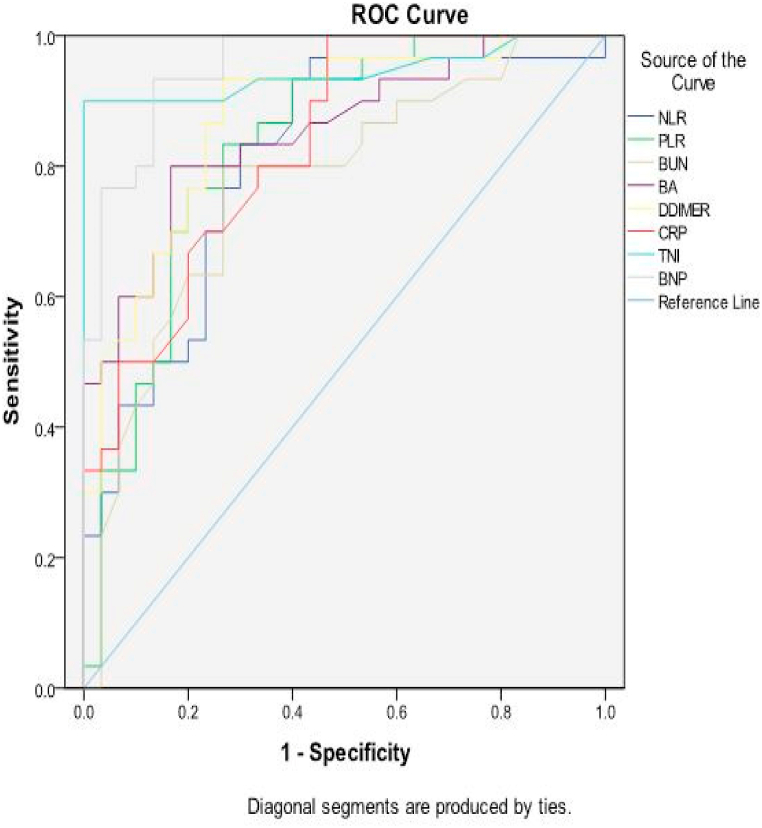
Receiver-operating characteristics (ROC) curve of NLR, PLR, BUN, BA ratio, D-dimer, CRP, TnI, BNP in predicting high risk groups among studied patients. N*LR* neutrophil/lymphocyte ratio, *PLR* platelet/lymphocyte ratio, *BUN* blood urea nitrogen, *B/A* BUN/albumin ratio, *CRP* C-reactive protein, *TnI* troponin I, *BNP* Brain natriuretic peptide.

Table 5 showed performance of serum biomarkers in predicting high risk groups among the studied patients. It indicated that TnI had the highest specificity (93.3 %) and PVP (93.1 %) with a cut-off value ≥ 0.21 ng/ml, area under the curve (AUC) 0.943, sensitivity and PVN were, 90 %, 90.3 % respectively. BNP had the highest sensitivity (93.3 %) and PVN (92.6 %) with a cut-off value ≥ 176 pg/ml, AUC 0.953, specificity and PVP were 83.3 %, 84.4 % respectively. The lowest sensitivity was demonstrated by Lymphocyte and NLR (70 %) with AUC of 0.768, 0.809 and specificity 70 %, 73.3 % respectively. The lowest specificity was recorded by albumin and B/A ratio (63.3 %) with AUC of 0.779, 0.853 and sensitivity 80 %, 83.3 % respectively. D-dimer had AUC 0.874, sensitivity 86.7 %, specificity 76.7 %, PVP 78.8 % and PVN 81.4 % with cut-off value ≥ 789.5 ng/ml. PLR, BUN and CRP showed good sensitivity (80 %, 80 %, 76.7 % respectively) but low specificity (73.3 %, 70 %, 66.7 % respectively) with AUC 0.831, 0.722, 0.831 respectively (Fig. 1, Fig. 2).

**Table 5:** Performance of serum biomarkers in predicting high risk groups among the studied patients (n = 60).

Variable	Cutoff-point	AUC	Sensitivity	Specificity	PVP	PVN	Accuracy
Lymphocytes (10³/ul):	≤1.3	0.809	70 %	70 %	70 %	70 %	70 %
Albumin (g/dl):	≤3.6	0.831	80 %	63.3 %	68.5 %	76 %	71.7 %
NLR	≥7.01	0.722	70 %	73.3 %	72.4 %	71 %	71.7 %
PLR	≥201.6	0.853	80 %	73.3 %	75 %	78.5 %	76.7 %
BUN (mg/dl):	≥20.4	0.874	80 %	70 %	72.7 %	77.8 %	75 %
B/A	≥5.3	0.831	83.3 %	63.3 %	69.4 %	79.2 %	73.3 %
CRP(mg/l):	≥9.0	0.943	76.7 %	66.7 %	69.7 %	74 %	71.7 %
D-Dimer (ng/ml):	≥789.5	0.953	86.7 %	76.7 %	78.8 %	81.4 %	81.7 %
Troponin I (ng/ml):	≥0.21	0.768	90 %	93.3 %	93.1 %	90.3 %	91.7 %
BNP (pg/ml):	≥176	0.779	93.3 %	83.3 %	84.8 %	92.6 %	88.3 %

*AUC* area under the ROC curve, *PVP* predictive value positive, *PVN* predictive value negative.

*NLR* neutrophil/lymphocyte ratio, *PLR* platelet/lymphocyte ratio, *BUN* blood urea nitrogen, *B/A* BUN/albumin ratio, *CRP* C-reactive protein, *BNP* Brain natriuretic peptide.

## 5 Discussion

Risk categorization of APE patients is crucial in order to permit evaluation patients' prognosis and direct the decision of management. Even though APE risk categorization approaches, a simple and easily applicable strategy to assess prognosis is still required [12].

Currently, APE severity and prognosis are evaluated using models for predicting risk like PESI and sPESI score to improve patient management. Only medical history and indicators of hemodynamic stability are taken into account by the present APE prediction models of mortality [13].

Presently, the main method for assessing risk in normotensive individuals is echocardiography. RV overload is acknowledged to be associated with an elevated mortality risk of up to 10 % in normotensives, despite that thrombolysis in sub-massive APE remains debatable. Additionally, echocardiography has a number of drawbacks. It depends on the personal efficiency, is not entirely replicable, and is not always immediately accessible [14].

A comprehensive risk categorization strategy incorporating clinical risk assessments, and/or imaging should ideally involve biomarkers [15]. A more accurate prognostic of death appears to be the concomitant existence of increased biomarkers and RV dysfunction on echocardiography [16].

So, we evaluated the efficacy of biomarkers in risk assessment of APE in comparison with the latest risk stratification methods which including haemodynamic measures and RV dysfunction echocardiographic markers.

This study was established aiming to evaluate the significance of serum biomarkers in APE risk assessment and the suitability of these biomarkers for management and decision-making.

No significant differences among our studied patients in baseline demographics or comorbidities.

In our study, several biomarkers (inflammatory, vascular and cardiac) that can be easily and quickly were assessed to evaluate their significance in risk assessment of APE patients.

We compared routine laboratory investigations (CBC, LFT, KFT, Bleeding profile) between categories of severity of APE patients. Regarding CBC, our study reported statistically non-significant differences among severity categories of PE patients as regards TLC, hemoglobin, hematocrit, neutrophils and PLT values, but statistically significant difference as regards lymphocytes (p = 0.001).

In consistence with our results, Haba et al. [17] found that there were statistically non-significant differences according to risk groups of PE regarding leukocytes, hemoglobin and thrombocytes. Also, Lee et al. [18] reported no statistical significant differences regarding PLT and hemoglobin between PE risk groups but statistically significant as regards TLC.

Kundi et al. [19] and Gok and Kurtul [20] revealed different results. Patients had highly statistically significant differences among severity groups according to TLC and PLT. The first study classified APE severity groups according to sPESI alone and the other study classified the severity groups to massive, submassive, and nonmassive. Also, Omar et al. [21] found that non-survivors group had significantly higher WBC compared with the survivors group during ICU hospitalization.

According to Slajus et al. [13], deceased patients had considerably reduced levels of hematocrit, haemoglobin, lymphocyte count, eosinophil count, and LMR. Although the platelet count was lower and the white blood cell and neutrophil counts were higher in deceased patients, there was no discernible difference.

According to **Sanchez et al.** [22]**,** the RV dysfunction caused by APE may result in an elevated TLC, which is a recognized marker of poor prognosis in APE patients.

Acute inflammation associated with APE leads to subsequent neutrophil recruitment and PLT activation and this has been linked to bad prognosis and rapid mortality. Furthermore, as a result of the release of glucocorticoids and adrenaline through sympathetic reaction, lymphocyte count also drops [20].

Lysosomal enzymes, histamine, leukotrienes, and other active chemicals are released when neutrophils accumulate up in the microvasculature, along with cytokines including IL-6, IL-10, and tumor necrosis factor (TNF). These cytokines and active chemicals can cause thrombosis through the stimulation of platelet adhesion and accumulation, vasoconstriction, and destroying endothelium and the vessel wall [23].

Regarding LFT and KFT, liver enzymes (ALT, AST) and s.creatinine showed statistically non-significant differences among severity categories of APE patients (p = 0.38, 0.85. 0.99 respectively). While regards s.albumin and BUN, there were statistically significant differences among studied groups (p = 0.001, 0.002 respectively).Similar results were reported by Omar et al. [21] who found that patients with massive APE had a significantly lower albumin level compared with subjects with non-massive APE. According to their findings, a life-threatening APE had a 75 % increased likelihood for every 1 gm/dL drop in albumin level. Hypoalbuminemia is an independent marker of death after PE, according to Hoskin et al. [24].

Serum albumin is essential for preserving physiological homeostasis as it helps colloid osmotic pressure maintains normal, improves arterial hyporeactivity, decreases ischemia reperfusion harm, and also acts as anti-inflammatory element [25]**.**

It can combine with nitric oxide to produce S-nitrosoproteins, which in turn stimulate vasomotor function and prevent platelet aggregation [26].

A compelling support to our results recorded by Tatlisu et al. [27] in the study involving 252 patients showed that BUN could detect high-risk patients with APE. Also, Omar et al. [21] revealed that the non-survivors group had significantly higher BUN and lower serum albumin than the survivors group.

A previous study documented the correlation between APE and the RV dysfunction. A condition of hypoperfusion can be reflected by BUN, which could be a marker of the RV systolic dysfunction that leads to renal hypoperfusion [28].

Also, hypoxemia and renal artery vasoconstriction due to disturbances of catecholamines may associate with renal impairment. Renal markers may offer an advantage over blood pressure in determining the severity of PE as they may more accurately reflect microcirculation dysfunction [29].

Inflammatory cytokines are released as a result of severe hypoxia accompanying PE and venous thromboembolism (VTE). Thus, inflammation has a significant impact in PE [30].

We evaluated the prognostic value of HHR, NLR, PLR, CRP and B/A ratio as inflammatory biomarkers, which are simple to evaluate indicators that have been proved to play a predictive value in APE.

HHR is the measure of hemoconcentration. Normal HHR in healthy people is 3, so if the HHR is higher than 3, the patient may be hemoconcentrated [31]. Very limited studies assessed the prognostic value of HHR in APE risk stratification.

Low haemoglobin and hematocrit levels were proven to be a reliable predictor of all-cause death in APE, according to Slajus et al. [13]. However our study showed no statistically significant differences among PE risk groups as regards hemoglobin, hematocrit, and HHR.

Recent research shown that NLR is a superior inflammatory marker to TLC [32]. The production of neutrophils and apoptosis of lymphocyte are accelerated by the inflammatory process [33].

In our study, there was highly statistically significant increase among severity categories of PE patients as regards NLR (p < 0.001) with statistically significant difference between low, intermediate low groups and intermediate high, high risk groups. Also, there was statistically significant increase among severity categories of APE patients as regards PLR (p = 0,005).

According to Kundi et al. [19]**,** since endothelial injury and inflammation both contribute to the development of APE, PLR may be a better predictor of death than platelet or lymphocyte counts separately.

Similar results were demonstrated by Kasapoğlu et al. [32] that NLR, PLR were significantly higher in non-survivors than survivors. Telo et al. [34] further showed that PLR and NLR were increased in high-risk PE patients. Also, Bi et al. [23] found that the levels of NLR in the death group were significantly higher than those in the survival group and Kundi et al. [19] revealed that PLR was highly significant increase in high risk than low risk group.

In a meta-analysis by Wang et al. [30]**,** the authors investigated the prognostic value of NLR and PLR in APE and found a significant correlation with short and long term prognosis. Other several studies reported similar findings about the predictive value of NLR and PLR in APE indicating that they could be routinely measured for prognostic evaluation [[35], [36], [37]].

Different results showed by Guzel et al. [38] that there were no difference between low sPESI and high sPESI groups in terms of NLR and PLR. They explained that they excluded a wider population of patients compared with other studies may be the reason of these different results.

PLR and NLR have the advantage of integrating information regarding inflammation and primary hemostasis [39].

Dehydration, malnutrition and liver and kidney reserve can all be taken into account when evaluating B/A as a comprehensive body reserve, which may be more helpful in determining the severity of an illness than BUN or serum albumin [40]. In our study, there was highly statistically significant increase in B/A ratio among severity groups of APE patients (p < 0.001).

The same finding proved by Fang and Xu [41] who reported highly statistically significant increase in non-survivors than survivors in patients with APE. They hypothesized that rather than BUN alone, the B/A ratio would be a more accurate predictor of death in APE patients.

Some researchers have evaluated the association between B/A and the prediction of patients with respiratory disorders. Ryu et al. [42] showed that BAR is a valuable prognostic element for aspiration pneumonia. Moreover, Cai et al. [43] revealed that the initial B/A ratio in patients with sepsis was related to in-hospital mortality.

CRP is the first protein defined as being positive for the acute phase and is a sensitive indicator of systemic inflammation. Interleukin 6 and monocyte chemoattractant protein 1 have been found to increase the propensity to thrombose in response to CRP [39]. Regarding CRP, our study proved statistically significant increase in PE risk groups (p = 0,002).

This is in accordance with Sagcan et al. [44] results that showed highly statistically significant increase in CRP among low, intermediate and high risk groups. Likewise, Ozcan et al. [45] reported that CRP significantly was lower in the survivor group compared to non-survivors.

Abul et al. [46] found that RV dysfunction was more frequent among the individuals with higher CRP. Patients were followed up for 30 days in Doğan et al. [47], and the CRP level in the early mortality group was higher than that in the living group.

These results were inconsistence with reports of other studies like lee et al. [18], Gok and Kurtul [20], Haba et al. [17] who reported that there was no statistical significant difference in CRP levels among APE severity groups.

Regarding cardiac biomarkers, our results revealed highly statistically significant increase in TnI and BNP among APE severity groups (P < 0.001) with significant difference between intermediate high and high risk groups.

This is in accordance with Lee et al. [18] who proved highly statistically significant higher TnI and BNP with higher adverse consequences in the intermediate high to high risk group than in the low to intermediate low risk group. Also, Bi et al. [23] reported the serum levels of BNP and TnI were significantly higher in the high risk group than the moderate risk group and higher in the death than the survival group.

Several studies showed near similar results as; Gok and Kurtul [20] and Haba et al. [17] revealed statistical significant increase in hyper sensitive TnI (hsTnI) and NT-proBNP between four risk groups. Izci et al. [48] and Sagcan et al. [44] showed highly statistically significant increase in NT-proBNP but no significant difference in troponin among risk groups.

The morphologies of echocardiography and CTpA are complex, making them challenging to manipulate. BNP, Tn, H-FABP, and imaging evidence of RVD and myocardial injury have a very strong correlation. These biomarkers show greater usefulness in predicting short-term prognosis compared to imaging findings [29].

Although they are not specific to VTE and can rise in a variety of clinical conditions, troponins and natriuretic peptides have the advantage of being easier to obtain than an echocardiogram or a CT scan. Elevated serum biomarkers of myocardial ischemia (Troponin I or Troponin T) or myocardial stretch (BNP- ProNBNP) show that the RV's adaptive mechanisms have failed and raise the possibility of hemodynamic instability [49].

Troponins, originally TnI and then hsTnT, were found to be indicators of the risk of sequelae 30 days after pulmonary embolism [50]. TnI levels rise as a result of decreased coronary blood flow, an abrupt increase in pulmonary artery and right ventricular pressure that may result in right ventricular dilatation, right ventricular myocardial ischemia, and even myocardial infarction [51].

Additionally, when pressure rises and the ventricular myocytes are abnormally stretched, a significant amount of BNP can be released into the blood. As a result, a high level of BNP can signify ventricular dysfunction and represent the severity of myocardial cell damage [52].

When compared to cardiac TN, HFABP diffuses much more quickly and manifests in the blood as early as 30 min after the symptoms onset. It is a rapid, extremely sensitive early indicator of myocardial damage [53]. According to ESC recommendations, HFABP offers prognostic data in acute PE, both in unselected and normotensive individuals [8].

In our study, 46 patients had positive H-FABP with no statistically significant difference among severity groups as regards H-FABP (p = 0.282).

The same result showed by Vuilleumier et al. [54] found that HFABP was not an independent prognostic factor in multivariate analysis. Also, Jenab et al. [55] revealed that association was found neither with the short-term adverse events nor long term mortality in terms of H-FABP.

Several studies reported different results; Boscheri et al. [56], Dellas et al. [57] and Qian et al. [53] indicated that positive H-FABP was an indicator of severity and prognosis of APE patients.

In our study, we measured D-dimer as one of vascular biomarkers. D-Dimer is a fibrin formation and degradation biomarker. For the diagnosis of APE, it has an adequate sensitivity and a strong negative predictive value. The predictive correlation between a high D-dimer and adverse outcomes has garnered a greater interest [3]. In our results, there was statistically significant increase in D-dimer among PE severity categories (P = 0.002).

This in accordance with Kasapoğlu et al. [32] who reported significant difference between the survivors and the non-survivors group regarding D-dimer. Similarly Bi et al. [23] and Gok and Kurtul [20] revealed that the levels of D-dimer were significantly higher in the high risk group than the moderate and low risk group and significant higher in the death than the survival group in the first study but no significant in the second.

On the other hand, Guzel et al. [38] Lee et al. [18], Izci et al. [48], Haba et al. [17] revealed no significant relation regarding D-Dimer in PE severity groups.

The ability of albumin to bind metals is changed in acute ischemia, leading to a metabolically altered protein with decreased metal binding capacity. This alteration is measurable and frequently recognized as ischemia modified albumin (IMA). It's recognized as a sign of oxidative stress [58].

IMA is a sensitive predictor in a variety of ischemic diseases, including myocardial, mesenteric ischemia and stroke, according to numerous studies [59]. In our study, we measured IMA and found statistically non-significant increase between PE severity groups (P = 0.864).

The majority of studies evaluated the role of IMA in the diagnosis of PE and found that IMA is a more sensitive marker than D-dimer in the detection of APE [60].

In the study of **Kaya et al.** [61]**,** the correlation between IMA and RV dysfunction was evaluated with no significant correlation between both. In another study performed by Türedi et al. [58] serum IMA levels were not correlated with pulmonary artery occlusion index (PAOI) and RV/LV ratio measured with pulmonary CTpA. Another study of Hogg et al. [62] showed that IMA levels are not specific for deaths related with PE.

On the contrary, Sagcan et al. [44] proved that there was highly statistically significant increase in IMA levels between APE severity groups. In this study, IMA was measured by different method than ours.

In this study TEE showed highly statistically significant increase among pulmonary embolism risk groups as regards RV/LV ratio (P value < 0.001) and statistically significant increase as regards PASP (P value = 0.003). In addition, there were highly statistically significant decrease as regards TAPSE and S′ (P value < 0.001).

This is consistent with the study done by Sanchez et al. [63] who concluded that the plasma concentrations of BNP and cTnI, and the RV/LV ratio increased significantly with the PESI.

In another study, TAPSE was lower in the patients with sPESI ≥1 than the sPESI <1 group. CST level was negatively correlated with TAPSE. In patients with sPESI ≥1, S’ was lower than the sPESI <1 group [48].

Also the study of El-Morshedy et al. [64] the studied cases were subdivided into two classes: Class I with intermediate-low-risk pulmonary embolism included 32 patients (53.3 %) and class II with intermediate high-risk pulmonary embolism involved 28 cases (46.7 %). Dyspnea, tachypnea, troponin level, RVD, RVD/LVD, and tricuspid regurgitation peak gradient to tricuspid annular plane systolic excursion (TRPG/TAPSE) were statistically higher in cases of class II than that of class I. On the other hand, TAPSE and PA acceleration time were markedly lower in the case of class II than that of class I. Ten cases (35.7 %) of group II required thrombolytic agents with a significant difference.

Ciurzyński et al. [65] stated that TAPSE and RV/LV ratio are useful to predict outcomes in non-elevated risk APE cases. This study set the cut-off point of TAPSE as >20 mm and this gives 100 % NPV for the CE. Therefore, cases with TAPSE >20 mm represent a least-risk class with a good prognosis.

There are many restrictions with echocardiography. It is user-dependent and not always immediately accessible.

In our study, despite of management of APE according to approved guidelines [8], 15 % of high risk group and 10 % of intermediate-high risk group patients died. 30 % of intermediate-high risk group, 20 % of intermediate-low risk and 10 % of low risk group patients deteriorated.

In order to effectively and appropriately manage this potentially fatal clinical entity, indicators other than hemodynamics that might reveal situations that require multimodal care due to poor prognosis are necessary.

These biomarkers' ideal cutoff values for risk categorization are uncertain. Even with the same assay, the investigations were quite heterogeneous in terms of assay method and cutoffs. Additionally, it is likely that reported variations in cut-off values utilising a similar technique are caused by small sample volumes and differences in the characteristics of patients [15].

In our study, the performance of serum biomarkers in predicting high risk groups among the studied patients was assessed. It indicated that TnI had the highest specificity and PVP among significant biomarkers followed by BNP. As regards sensitivity and PVN, BNP was the highest followed by TnI. Also, D-dimer showed high sensitivity and specificity in predicting high risk groups.

Measurement of troponin was proved to increase the echocardiography predictive significance of in APE and the incorporation of biomarkers into the echocardiography-based risk evaluation approaches is well-established [15].

According to ESC recommendations, low levels of BNP or NT-proBNP have a high degree of sensitivity and a negative predictive value for the ability to rule out a poor early clinical prognosis [8].

In order to investigate the predictive effectiveness of cardiac biomarkers in elderly people in comparison with clinical scores, Vuilleumier et al. [66] examined a total of 230 patients who aged ≥65 years old with non-high risk APE. The researchers discovered that at least in the elderly, assessing hsTnT or NT-pro BNP is at least equally valuable for prognostication as the PESI score.

Thus, biomarker testing completes prognostic data. Patients who have elevated levels of cardiac biomarkers, notably a positive troponin and imaging signs of RV dysfunction should therefore be categorized as being at intermediate-high risk. These patients need to be closely observed for the first couple of days in order to detect any hemodynamic decompensation and start rescue reperfusion therapy without delay. Additionally, low risk patients are transformed into intermediate-low risk patients by either an imaging test or a positive biomarker [15].

Owing to the progressing of a thrombus' cellular and inflammatory characteristics, it might be beneficial to evaluate laboratory results at different times. In addition to the possibility to the predicting mortality risk using blood cells markers at a later time point, but stronger prognostic capacity might be revealed by examining laboratory responsiveness to therapies [13].

Our study had some limitations. The study had a relatively small number of participants and only involved one Centre. The small sample size is due to the study's stringent inclusion and exclusion criteria as well as the challenging task of gathering clinical cases.

## 6 Conclusion

APE prognosis can be judged accurately by simultaneously measuring a few biomarkers along with haemodynamic variables and echocardiographic parameters of RV dysfunction. Due to the assays' low cost, limited degree of invasiveness, and wide availability, measuring these biomarkers is particularly helpful in actual medical practice.

## Declarations

Ethics approval and Consent to Participate:

This study was approved by institutional board review of Zagazig University **(ZU-IRB #10144/28**–**12**–**2022)** Patients enrolled in this study provided their informed and signed consent.

## Funding

There was no specific funding for this study from public, private, or non-profit funding sources.

## Consent for publication

Not available.

## Availability of data and materials

The corresponding author can give the database that was used and examined in this study upon reasonable request.

## Authors’ contributions

HAE Writing the manuscript, Practical part of the study. NEZ Revising the manuscript. MAA Practical part of the study. MS Practical part of the study. ATE Practical part of the study. MMN Selecting the study subject, Revising the manuscript. All authors have read and accepted the manuscript.

## Declaration of competing interest

The authors declare that they have no known competing financial interests or personal relationships that could have appeared to influence the work reported in this paper.
